# Empagliflozin to prevent worsening of left ventricular volumes and systolic function after myocardial infarction (EMPRESS‐MI)

**DOI:** 10.1002/ejhf.3560

**Published:** 2024-12-15

**Authors:** Jaclyn Carberry, Mark C. Petrie, Matthew M.Y. Lee, Bethany Stanley, Katriona J.M. Brooksbank, Ross T. Campbell, Richard Good, Pardeep S. Jhund, Peter Kellman, Ninian N. Lang, M. Mitchell Lindsay, Kenneth Mangion, Roy S. Gardner, Patrick B. Mark, Barbara Meyer, Joanne O'Donnell, Vanessa Orchard, Aadil Shaukat, Stuart Watkins, Alex McConnachie, John J.V. McMurray, Paul Welsh, Naveed Sattar, Colin Berry, Kieran F. Docherty

**Affiliations:** ^1^ British Heart Foundation Glasgow Cardiovascular Research Centre University of Glasgow Glasgow UK; ^2^ Golden Jubilee National Hospital Clydebank UK; ^3^ Robertson Centre for Biostatistics, School of Health and Wellbeing University of Glasgow Glasgow UK; ^4^ West of Scotland Innovation Hub, National Health Service Scotland Glasgow UK; ^5^ National Heart, Lung, and Blood Institute, National Institutes of Health Bethesda MD USA

**Keywords:** Heart failure, Left ventricular remodelling, Myocardial infarction, SGLT2 inhibitors

## Abstract

**Aims:**

Patients with a reduced left ventricular ejection fraction (LVEF) following an acute myocardial infarction (MI) are considered to be at risk of progressive adverse cardiac remodelling which can lead to the development of heart failure and death. The early addition of a sodium–glucose cotransporter 2 (SGLT2) inhibitor to standard treatment may delay or prevent progressive adverse remodelling in these patients.

**Methods and results:**

We performed a randomized, double‐blind, placebo‐controlled, multicentre trial using cardiovascular magnetic resonance imaging (MRI), in patients with left ventricular systolic dysfunction following MI. Eligible patients were those ≥12 h and ≤14 days following acute MI, with an LVEF <45% by MRI. Patients were randomized to empagliflozin 10 mg once a day or matching placebo. The primary outcome was the change in left ventricular end‐systolic volume indexed to body surface area (LVESVI) from baseline to 24 weeks. Secondary outcomes included measures of left ventricular and atrial volumes, left ventricular mass, LVEF, and high‐sensitivity troponin I (hs‐TnI) and N‐terminal prohormone of B‐type natriuretic peptide (NT‐proBNP) concentrations. From October 2022 to January 2024, 105 eligible patients were randomized. The mean age was 63 ± 11 years and 90 (87%) were male. The mean LVEF was 34.8 ± 6.0%. In the placebo group, LVESVI decreased by 7.8 ± 16.3 ml/m^2^, left ventricular end‐diastolic volume index (LVEDVI) did not change (−0.3 ± 18.7 ml/m^2^) and LVEF increased by 8.5 ± 7.4% at 24 weeks from baseline. Empagliflozin did not affect the change in LVESVI from baseline to 24 weeks (between‐group difference = 0.3 ml/m^2^, 95% confidence interval −5.2 to 5.8; *p* = 0.92). Compared with placebo, empagliflozin also had no effect on LVEDVI, LVEF, left atrial volume index, left ventricular mass index, NT‐proBNP, or hs‐TnI, but did increase haematocrit and reduced uric acid and weight.

**Conclusions:**

In patients with left ventricular systolic dysfunction after an acute MI receiving contemporary standard of care, treatment with empagliflozin had no effect on cardiac volumes or LVEF compared with placebo. Progressive adverse cardiac remodelling did not occur in the majority of patients.

## Introduction

The development of heart failure following an acute myocardial infarction (MI) is associated with substantial morbidity and mortality.^1^, ^2^, ^3^ As such, the prevention of heart failure is an important therapeutic goal. Progressive adverse ventricular remodelling, characterized by ventricular dilatation and declining systolic function, is the precursor to heart failure after MI.^4^ Early reperfusion therapy and some of the drugs that reduce the risk of mortality and heart failure following MI prevent progressive adverse remodelling (e.g. angiotensin‐converting enzyme [ACE] inhibitors, angiotensin receptor blockers [ARB], and beta‐blockers).^4^, ^5^, ^6^, ^7^, ^8^, ^9^, ^10^, ^11^, ^12^, ^13^, ^14^ The mineralocorticoid receptor antagonist (MRA) eplerenone improved outcomes following MI, however was found to have a significant anti‐remodelling effect only after adjustment for baseline covariates.^15^, ^16^ Sacubitril/valsartan did not reduce the risk of incident heart failure or cardiovascular mortality in high‐risk patients following MI and had a minimal anti‐remodelling effect.^17^, ^18^

Sodium–glucose cotransporter 2 (SGLT2) inhibitors reduce the risk of worsening heart failure and mortality in patients with chronic heart failure across the full spectrum of left ventricular ejection fraction (LVEF).^19^ One of the mechanisms of action underlying the clinical benefits of SGLT2 inhibitors in heart failure with reduced ejection fraction (HFrEF) is a beneficial remodelling effect.^20^, ^21^, ^22^ Recently, the SGLT2 inhibitor empagliflozin did not reduce the risk of the primary outcome of heart failure hospitalization or all‐cause mortality in high‐risk patients following acute MI.^23^ However, empagliflozin did reduce the risk of first and total heart failure hospitalizations and adverse heart failure events.^24^ Whether this is related to a beneficial remodelling effect is unknown. In lower risk post‐MI patients, dapagliflozin improved cardiometabolic outcomes, but not the composite of cardiovascular death or heart failure hospitalization.^25^

We designed the EMpagliflozin to PREvent worSening of left ventricular volumes and Systolic function after Myocardial Infarction (EMPRESS‐MI) randomized, placebo‐controlled trial to test the hypothesis that empagliflozin, in addition to standard of care, would attenuate adverse left ventricular remodelling measured using cardiovascular magnetic resonance imaging (MRI) in high‐risk patients following acute MI.

## Methods

The design and methods of the EMPRESS‐MI trial have been published previously.^26^

### Trial organization and sources of funding

EMPRESS‐MI was conceived and designed by the Trial Steering Committee. The trial was co‐sponsored by the University of Glasgow and the National Health Service (NHS) Greater Glasgow and Clyde Health Board and registered at ClinicalTrials.gov (NCT05020704). The lead site was the NHS Golden Jubilee National Hospital. Funding for the trial and provision of empagliflozin and a matching placebo was provided by Boehringer Ingelheim. Boehringer Ingelheim had no role in the trial design, or trial conduct, and was not involved in data analysis or interpretation. The trial protocol and any substantial amendments were approved by the Newcastle Research Ethics Committee (22/NE/0030). The first participant was randomized on 6 October 2022.

### Eligibility

Eligible patients were men or women ≥18 years of age, who had a type 1 MI, i.e. an ST‐elevation MI (STEMI) or non‐STEMI (according to the fourth universal definition of MI), and an LVEF ≤40% measured by echocardiography.^27^ Patients were eligible for randomization within 12 h and 14 days of MI. Key exclusion criteria were a history of HFrEF, contraindications to SGLT2 inhibitors (a history of type 1 diabetes mellitus, ketoacidosis, allergy to SGLT2 inhibitors, current or planned use of SGLT2 inhibitors, or active genital infection). Patients with estimated glomerular filtration rate (eGFR) <30 ml/min/1.73 m^2^ (measured using the Modification of Diet in Renal Disease formula) were also excluded. Patients with permanent or persistent atrial fibrillation or an implanted cardiac device were excluded to avoid potential MRI image degradation. The complete inclusion and exclusion criteria are listed in online supplementary *Table* *S1*.

### Screening

Admission transthoracic echocardiograms, performed ≥12 h from admission as part of routine clinical care, were reviewed to identify patients with LVEF ≤40%, measured by Simpson's biplane or estimated by visual assessment. Those who met the inclusion and exclusion criteria were approached for consent. Consenting patients then had a baseline cardiovascular MRI scan. Patients with an LVEF <45% by MRI proceeded to randomization (changed from an LVEF ≤40% by an amendment to the trial protocol on 23 February 2023), and those with LVEF ≥45% were excluded from randomization.

### Randomization and stratification

Patients were randomized in a 1:1 ratio to receive either empagliflozin 10 mg once daily, or matching placebo. The randomization schedule was computer‐generated by the method of randomized permuted blocks, with random block lengths of 4 and 6. Randomization was stratified by MRI‐measured left ventricular end‐systolic volume indexed to body surface area (BSA) (LVESVI) (≤45 ml/m^2^/>45 ml/m^2^), the prescription of loop diuretics at the time of randomization, and the presence of type 2 diabetes mellitus (either an established diagnosis or a glycated haemoglobin [HbA1c] ≥48 mmol/mol at the index admission). Before randomization, baseline assessments were performed, including body weight and height and a EuroQol 5‐Dimension 5‐Level (EQ‐5D‐5L) questionnaire. Standard laboratory blood tests including kidney function, HbA1c and a full blood count, and blood and urine were collected for biomarker analysis. Randomization and administration of the first dose of the trial drug took place within 24 h of the baseline MRI. All participants and trial staff were blinded to treatment allocation.

### Study visits

Following randomization, trial visits took place at 2, 12, 18 and 24 weeks. Visit 2 (at 2 weeks) and visit 4 (at 18 weeks) were carried out by a telephone call. All other visits were in person. The schedule of assessments is detailed in online supplementary *Table* *S2*.

### Primary outcome

The primary outcome was the change in LVESVI from baseline to 24 weeks as measured by cardiovascular MRI.

### Secondary outcomes

The secondary outcomes, measured as change from baseline to 24 weeks, were: left ventricular end‐diastolic volume indexed to BSA (LVEDVI), LVEF, left atrial volume indexed to BSA (LAVI), left ventricular mass indexed to BSA (LVMI), N‐terminal prohormone of B‐type natriuretic peptide (NT‐proBNP), high‐sensitivity cardiac troponin I (hs‐TnI), and infarct size measured using cardiovascular MRI.

### Exploratory outcomes

Exploratory outcomes included the change in biomarkers relevant to the actions of empagliflozin (uric acid, HbA1c, and haematocrit), kidney function, and body weight. The change in patient‐reported health status measured using the EQ‐5D‐5L questionnaire was also analysed.

The proportion of participants with adverse left ventricular remodelling at 24 weeks was also analysed, defined as a ≥12% increase in LVEDVI only, or a ≥12% increase in both LVESVI and LVEDVI.^28^

### Cardiovascular magnetic resonance imaging acquisition

Cardiovascular MRI was performed at baseline and 24 weeks following randomization on a single 1.5 T Siemens MAGNETOM Avanto scanner. The imaging protocol included steady‐state free precession cine imaging, native and post‐contrast T1 mapping, T2 and T2* mapping, and late gadolinium enhancement imaging. The baseline and 24‐week scans were analysed in pairs to reduce intra‐observer variability, and the operator was blinded to patient identity and treatment allocation. Further details regarding the cardiovascular MRI protocol are available in the design paper.^26^ Cardiovascular MRI analysis methods are detailed in online supplementary material.

### Biomarkers

Glycated haemoglobin and haematocrit were measured as part of routine care in NHS Golden Jubilee National Hospital biochemistry labs. NT‐proBNP (Roche e411, Roche Diagnostics, Burgess Hill, UK), hs‐TnI (i1000SR ARCHITECT, Abbott Laboratories, Maidenhead, UK), uric acid and creatinine for eGFR (Roche c311, Roche Diagnostics, Burgess Hill, UK) were batch measured after study completion in stored blood samples taken at baseline, 12 weeks and 24 weeks. All non‐routine assays were performed at a central lab (GlasBRU, University of Glasgow) using the manufacturers' calibrators and quality controls.

### Statistical analysis

Statistical analyses were conducted at the study data centre (Clinical Trials Unit, Robertson Centre for Biostatistics, University of Glasgow) according to a pre‐specified statistical analysis plan. Efficacy analyses were performed according to the intention‐to‐treat principle, including all randomly assigned participants without major protocol deviations, and with post‐randomization data available for the outcome of interest at any given time point, irrespective of their subsequent participation in the study and their adherence to randomized treatment. The safety analysis included all randomized patients who took at least one dose of study medication. The sample size was 120 patients based on the calculation that 50 patients in each group would provide >90% power (α level = 0.05) to detect a mean between‐group difference in change in LVESVI from baseline of 6 ml/m^2^ (standard deviation [SD] of change = 7.8 ml/m^2^), and allowing for a 10% screen failure rate for LVEF and a 10% drop out rate for loss to follow‐up and death.^29^ Data were summarized descriptively for each randomized treatment group, using counts and percentages for categorical variables, and mean, SD, or median, interquartile range (IQR), depending on the distribution of the data. Each outcome was analysed using a linear regression analysis model adjusted for the randomized treatment group, the baseline value of the outcome in question, diuretic use at baseline, and diabetes status. The regression model treatment effect estimates were reported with 95% confidence intervals (CI) and *p*‐values. A *p*‐value of <0.05 was considered statistically significant. All analyses were conducted using R Studio and R version 4.4.1 (R Foundation for Statistical Computing, Vienna, Austria).

## Results

Recruitment took place between October 2022 and January 2024. Follow‐up visits were completed in June 2024. Of 131 patients screened, 105 were randomly assigned (51 to empagliflozin and 54 to placebo). The reasons for screen failures are detailed in the CONSORT diagram (online supplementary *Figure* *S1*). Following randomization, one participant in the placebo group had a diagnosis of cardiac amyloid and was excluded from the efficacy analysis.

### Baseline characteristics

The baseline characteristics summarized by randomized treatment allocation are displayed in *Table* *1*. The mean age was 63.0 ± 11.2 years, and 90 (87%) patients were male. The median time from MI to randomization was 3.0 days (IQR 2.0–5.0). Ninety‐two (88%) patients had a STEMI and 12 (12%) had an NSTEMI; 83 (80%) MIs were in the anterior location.

**Table 1:** Baseline characteristics

Characteristic	Empagliflozin (*n* = 51)	Placebo (*n* = 53)
Age, years	63.4 (10.8)	62.6 (11.7)
Male sex, *n* (%)	44 (86.3)	46 (86.8)
Systolic blood pressure, mmHg	114.6 (16.5)	113.9 (17.5)
Heart rate, bpm	77.9 (13.0)	73.5 (13.8)
eGFR^a^, ml/min/1.73 m^2^	78.3 (20.3)	79.3 (20.2)
Peak troponin T^b^, ng/L	5585 (2604–8132)	4514 (2578–7884)
NT‐proBNP, pg/ml	2316 (1110–3375)	2017 (1286–3175)
MRI LVEF, %	33.7 (6.0)	35.8 (5.8)
Myocardial infarction		
Time from symptom onset to reperfusion (STEMI patients only), min	431 (171–1339)	280 (163–550)
MI type, *n* (%)		
STEMI	46 (90.2)	46 (86.8)
NSTEMI	5 (9.8)	7 (13.2)
Infarct location, *n* (%)		
Anterior	41 (80.4)	42 (79.2)
Inferior	6 (11.8)	9 (17.0)
Lateral	4 (7.8)	2 (3.8)
PCI or thrombolysis, *n* (%)	50 (98.0)	53 (100)
PCI	49 (96.1)	53 (100)
Thrombolysis	2 (3.9)	3 (5.7)
Medical history, *n* (%)		
Hypertension	20 (39.2)	15 (28.3)
Diabetes	4 (7.8)	5 (9.4)
Stroke	1 (2.0)	2 (3.8)
Medications at randomization, *n* (%)		
Dual antiplatelet therapy	51 (100)	53 (100)
Anticoagulant	3 (5.9)	6 (11.3)
Statin	50 (98.0)	53 (100)
ACE inhibitor or ARB	50 (98.0)	47 (88.7)
Beta‐blocker	42 (82.4)	47 (88.7)
Mineralocorticoid receptor antagonist	33 (64.7)	33 (62.3)
Loop diuretic	15 (29.4)	15 (28.3)
Loop diuretic at any point from admission to randomization, *n* (%)	22 (43.1)	24 (45.3)

Data are presented as mean (standard deviation), or median (interquartile range) unless otherwise stated. Baseline characteristics are presented for all randomized patients included in the efficacy analysis.

ACE, angiotensin‐converting enzyme; ARB, angiotensin receptor blocker; eGFR, estimated glomerular filtration rate; IQR, interquartile range; LVEF, left ventricular ejection fraction; MI, myocardial infarction; MRI, magnetic resonance imaging; NSTEMI, non‐ST‐elevation myocardial infarction; NT‐proBNP, N‐terminal pro‐B‐type natriuretic peptide; PCI, percutaneous coronary intervention; SD, standard deviation; STEMI, ST‐elevation myocardial infarction.

^a^ Estimated glomerular filtration rate was calculated using the Modification of Diet in Renal Disease formula.

^b^ Available in 86 (83%) patients.

Nearly all patients (103 [99%]) had percutaneous coronary intervention (PCI) or thrombolysis; 102 (98%) had PCI performed and 5 (5%) received thrombolytic therapy. Of those with a STEMI, 86 (93%) had primary PCI with a median time from symptom onset to PCI of 5.6 h (IQR 2.8–19.4). At randomization, 97 (93%) patients were taking an ACE inhibitor or an ARB, 89 (86%) a beta blocker, 66 (63%) an MRA, and 30 (29%) were on a loop diuretic; 46 (44%) patients received a loop diuretic at any point during the index admission before randomization. Medication use during trial follow‐up is detailed in online supplementary *Table* *S3*. Symptoms and signs of heart failure at follow‐up are presented in online supplementary *Table* *S4*.

The mean LVEF by echocardiography was 35.0 ± 4.9% and by MRI was 34.8 ± 6.0%. The mean infarct size as a percentage of myocardial mass was 36.3 ± 13.4%. Microvascular obstruction was present in 77 (74%) patients and intramyocardial haemorrhage in 47 of the 97 with interpretable T2* maps (48%). The median baseline NT‐proBNP was 2109 pg/ml (IQR 1128–3375). The median peak troponin T was 4853 ng/L (IQR 2570–7945) (available in 86 [83%] patients).

### Completeness of follow‐up and adherence

Of the 51 patients randomized to empagliflozin, 49 remained on randomized therapy and 48 had complete primary outcome data at baseline and 24 weeks (online supplementary *Figure* *S1*). The one patient who did not have complete primary outcome data received an implantable cardioverter‐defibrillator following randomization, therefore did not attend for follow‐up MRI but remained on randomized treatment and attended for all other outcome data collection. Of the 53 patients randomized to placebo, 52 remained on randomized therapy and had complete primary outcome data at baseline and 24 weeks. The two patients (one in each treatment group) who discontinued randomized treatment before at least 12 weeks of treatment exposure did not undergo follow‐up MRI as per the trial protocol. There was one death (sudden cardiac death) in the empagliflozin group. There were no deaths in the placebo group.

### Primary outcome

**Figure 1:**
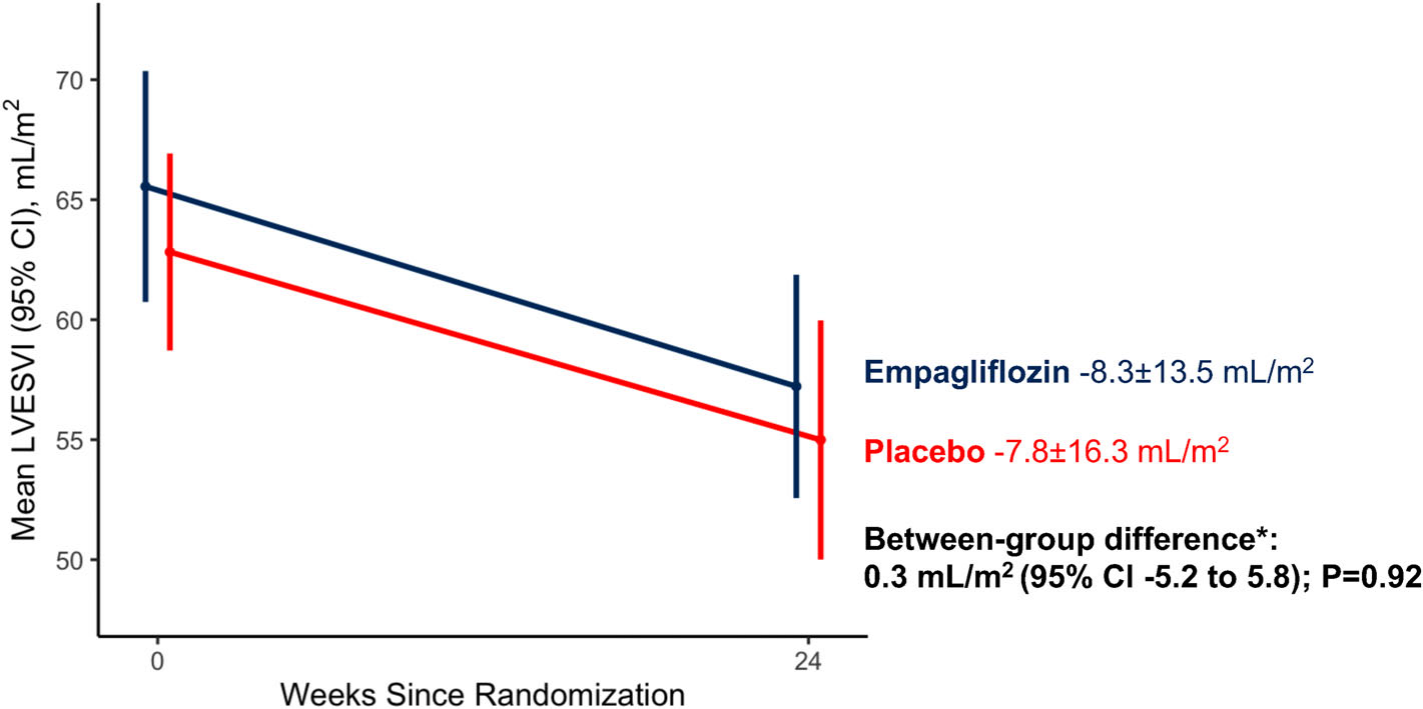
Change in left ventricular end‐systolic volume index (LVESVI) from baseline at 24 weeks. Data presented as mean and error bars represent 95% confidence intervals (CI). *Calculated using a linear regression model adjusted for randomized treatment, baseline value of the outcome, use of diuretics at baseline and diabetes status.

Empagliflozin had no effect compared with placebo on the change in LVESVI from baseline at 24 weeks. LVESVI decreased by 8.3 ± 13.5 ml/m^2^ between baseline and 24 weeks in the empagliflozin group and by 7.8 ± 16.3 ml/m^2^ in the placebo group. The adjusted between‐group difference in change in LVESVI was 0.3 ml/m^2^ (95% CI −5.2 to 5.8; *p* = 0.92) (*Table* *2* and *Figure* *1*).

**Table 2:** Change in primary and secondary outcomes with empagliflozin or placebo from baseline at 24 weeks

	Empagliflozin				Placebo				Between‐group difference (95% CI)^a^	*p*‐value
	*n*	Baseline	24 weeks	Change	*n*	Baseline	24 weeks	Change		
Primary outcome										
LVESVI, ml/m^2^	48	65.6 (17.0)	57.2 (16.5)	−8.3 (13.5)	52	62.8 (15.1)	55.0 (18.3)	−7.8 (16.3)	0.3 (−5.2, 5.8)	0.92
Secondary outcomes										
LVEDVI, ml/m^2^	48	97.8 (19.8)	98.3 (17.3)	0.6 (16.3)	52	97.6 (18.3)	97.3 (22.2)	−0.3 (18.7)	0.8 (−5.5, 7.0)	0.81
LVEF, %	48	33.4 (6.0)	42.7 (8.6)	9.4 (7.5)	52	36.0 (5.8)	44.4 (7.6)	8.5 (7.4)	0.0 (−2.9, 3.0)	0.98
LAVI, ml/m^2^	48	34.3 (12.7)	37.3 (13.4)	3.0 (14.4)	52	36.2 (11.2)	39.2 (13.0)	3.0 (13.3)	−1.0 (−5.9, 3.8)	0.67
LVMI, g/m^2^	48	62.2 (13.7)	52.2 (9.6)	−10.0 (7.8)	52	59.1 (11.0)	50.4 (9.4)	−8.7 (6.4)	−0.3 (−2.4, 1.8)	0.78
NT‐proBNP, pg/ml	49	2316 (1075, 3413)	346 (182, 822)	−1794 (−2763, −916)	52	2017 (1286, 3157)	387 (170, 759)	−1486 (−2529, −799)	0.95 (0.68, 1.34)	0.77
hs‐TnI, ng/L	50	17 552 (8417, 34 473)	8 (5, 15)	−17 547 (−34 362, −8408)	53	16 047 (6529, 31 040)	7 (5, 14)	−16 040 (−31 033, −6511)	1.10 (0.81, 1.50)	0.54
Infarct size, %	47	37.9 (11.8)	28.7 (10.7)	−9.1 (7.7)	49	33.9 (15.2)	24.0 (13.1)	−9.9 (9.3)	2.1 (−0.9, 5.2)	0.16

Data are presented as mean (standard deviation), or median (interquartile range) unless otherwise stated. Results are reported for those with data available at baseline and 24 weeks.

CI, confidence interval; hs‐TnI, high‐sensitivity cardiac troponin I; LAVI, left atrial volume index; LVEDVI, left ventricular end‐diastolic volume index; LVEF, left ventricular ejection fraction; LVESVI, left ventricular end‐systolic volume index; LVMI, left ventricular mass index; NT‐proBNP, N‐terminal prohormone of B‐type natriuretic peptide.

^a^ Calculated using a linear model adjusted for randomized treatment, baseline value of the outcome, use of diuretics at baseline and diabetes status. Between‐group differences are reported as ratios of adjusted geometric means for NT‐proBNP and hs‐TnI from models using log‐transformed values. All other outcomes are reported as adjusted mean differences (95% CI).

### Secondary outcomes

#### Cardiovascular magnetic resonance imaging

**Figure 2:**
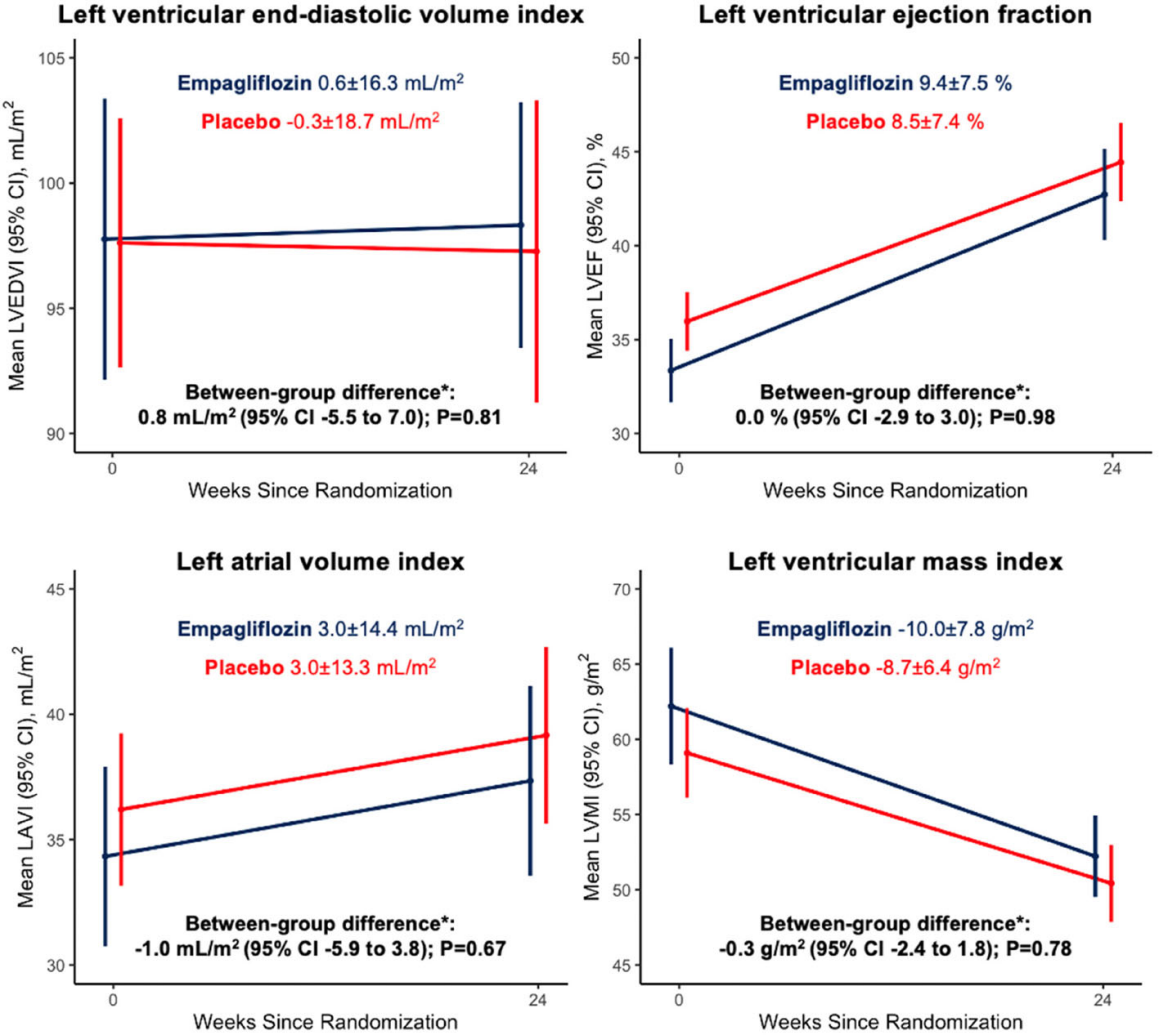
Change in secondary cardiovascular magnetic resonance imaging outcomes from baseline at 24 weeks. Data presented as mean and error bars represent 95% confidence intervals (CI). LAVI, left atrial volume index; LVEDVI, left ventricular end‐diastolic volume index; LVEF, left ventricular ejection fraction; LVESVI, left ventricular end‐systolic volume index; LVMI, left ventricular mass index. *Calculated using a linear regression model adjusted for randomized treatment, baseline value of the outcome, use of diuretics at baseline and diabetes status.

Empagliflozin had no effect on any of the MRI secondary endpoints. The mean change in LVEDVI in the empagliflozin group was 0.6 ± 16.3 ml/m^2^ and −0.3 ± 18.7 ml/m^2^ in the placebo group between baseline and 24 weeks (adjusted between‐group difference 0.8 ml/m^2^; 95% CI −5.5 to 7.0; *p* = 0.81) (*Table* *2* and *Figure* *2*). LVEF increased by 9.4 ± 7.5% and 8.5 ± 7.4% in the empagliflozin and placebo groups, respectively, with no between‐group difference (0.0%; 95% CI −2.9 to 3.0; *p* = 0.98) (*Table* *2*, *Figure* *2* and online supplementary *Table* *S5*). LAVI increased in both groups, and LVMI and infarct size decreased in both groups, with no between‐group difference in any measure (*Table* *2* and *Figure* *2*). A post‐hoc analysis of non‐indexed MRI values yielded similar results (online supplementary *Table* *S6*).

#### NT‐proBNP and troponin

NT‐proBNP and hs‐TnI decreased in both the empagliflozin and placebo groups between baseline and 24 weeks (*Table* *2*). There was no between‐group difference in either biomarker (*Table* *2*).

### Exploratory outcomes

Empagliflozin, compared to placebo, decreased uric acid (*p* = 0.006) and increased haematocrit (*p* = 0.006) (*Table* *3*). There were no between‐group differences for the other measured biomarkers (*Table* *3*).

**Table 3:** Change in exploratory outcomes with empagliflozin or placebo from baseline at 24 weeks

	Empagliflozin				Placebo				Between‐group difference (95% CI)^a^	*p*‐value
	*n*	Baseline	24 weeks	Change	*n*	Baseline	24 weeks	Change		
Uric acid, μmol/L	49	5.6 (4.6, 6.6)	4.7 (4.0, 6.3)	−0.6 (−1.9, 0.2)	53	5.6 (4.5, 6.6)	5.6 (4.4, 6.4)	−0.1 (−0.9, 1.3)	0.85 (0.75, 0.95)	0.006
HbA1c, mmol/mol	50	39.6 (11.6)	40.0 (5.1)	0.4 (10.0)	53	39.3 (7.7)	40.0 (6.1)	0.7 (3.4)	−0.1 (−1.7, 1.6)	0.94
eGFR, ml/min/1.73 m^2^	50	78.5 (20.5)	73.6 (17.5)	−4.8 (13.0)	53	79.3 (20.2)	72.3 (17.0)	−7.1 (15.1)	1.8 (−2.8, 6.5)	0.42
Haematocrit, L/L	48	0.40 (0.05)	0.42 (0.05)	0.02 (0.05)	52	0.41 (0.05)	0.40 (0.05)	−0.01 (0.04)	0.02 (0.01, 0.04)	0.006
Body weight, kg	48	84.0 (19.5)	81.5 (18.2)	−2.5 (6.0)	52	82.0 (15.6)	82.5 (17.1)	0.5 (4.2)	−2.9 (−5.0, −0.9)	0.006
EQ‐5D‐5L visual analogue scale	50	67.6 (21.2)	79.6 (13.5)	12.0 (21.5)	53	62.2 (22.6)	76.4 (19.5)	14.2 (25.0)	2.2 (−4.2, 8.6)	0.50
EQ‐5D‐5L total index score	49	0.77 (0.25)	0.86 (0.15)	0.09 (0.22)	53	0.72 (0.29)	0.82 (0.19)	0.10 (0.30)	0.03 (−0.04, 0.09)	0.38

Data are presented as mean (standard deviation), or median (interquartile range) unless otherwise stated. Results reported for those patients with data available at baseline and 24 weeks.

CI, confidence interval; eGFR, estimated glomerular filtration rate; EQ‐5D‐5L, EuroQol 5‐Dimension 5‐Level; HbA1c, glycated haemoglobin.

^a^ Calculated using a linear regression model adjusted for randomized treatment, baseline value of the outcome, use of diuretics at baseline and diabetes status. Between‐group differences are reported as ratios of adjusted geometric means for uric acid from models using log‐transformed values. All other outcomes are reported as adjusted mean differences (95% CI).

Empagliflozin, compared to placebo, reduced body weight with a between‐group difference of −2.9 kg (95% CI −5.0 to −0.9; *p* = 0.006) (*Table* *3*).

Empagliflozin, compared to placebo, did not change patient‐reported health status, measured by EQ‐5D‐5L (*Table* *3*).

### Safety

There were no between‐group differences in safety outcomes (online supplementary *Table* *S7*). There were few cases of genital infections (*n* = 3 in the empagliflozin group, *n* = 1 in the placebo group). This required permanent discontinuation of study treatment in two cases (both in the empagliflozin group). There was one case of worsening kidney function (≥2‐fold increase in creatinine) which occurred in the placebo group. There was no between‐group difference in systolic blood pressure between baseline and 24 weeks (online supplementary *Table* *S8*).

## Discussion

In patients with left ventricular systolic dysfunction after an acute MI, empagliflozin, compared with placebo, did not change left ventricular volumes or systolic function over 24 weeks of treatment.

Sodium–glucose cotransporter 2 inhibitors reduce the risk of worsening heart failure and mortality in patients with HFrEF.^19^ One of the mechanisms of these benefits is a favourable effect on adverse remodelling that has been reported in three small randomized controlled trials.^20^, ^21^, ^22^ The hypothesis of the EMPRESS‐MI trial was that the remodelling benefits seen with SGLT2 inhibitors in patients with established HFrEF would extend to patients at high risk of developing heart failure as a result of an acute MI complicated by left ventricular systolic dysfunction.

One key aspect of our hypothesis deserves further consideration. We designed the EMPRESS‐MI trial to enrol patients that we believed to be at a high risk of progressive adverse remodelling to test whether empagliflozin would attenuate or prevent this process. To try and exclude patients with ventricular ‘stunning’, the natural history of which is the rapid recovery of systolic dysfunction, the eligibility criteria stipulated that patients should have a depressed LVEF on both transthoracic echocardiography at least 12 h following MI and on MRI before randomization.^30^ Despite this, the trajectory of change in left ventricular volumes and systolic function in both the empagliflozin and placebo groups was one of improvement, i.e. we did not see progressive adverse remodelling in the overall trial population. In the placebo group at 24 weeks, the mean reduction in LVESVI from baseline was 7.8 ml/m^2^ and the mean increase in LVEF was 8.5%. LVEDVI did not change during follow‐up but this is in the context of a reduction in heart rate of 10 bpm from randomization to 24 weeks which would otherwise be expected to be associated with an increase in LVEDVI. Only a small proportion of patients had evidence of progressive ventricular enlargement; LVEDVI alone increased by ≥12% in 15 patients (15%) and both LVEDVI and LVESVI increased by ≥12% in 13 (13%).^28^ This would suggest that in the context of a median time from MI to randomization of 3 (IQR 2–5) days, the majority of the cohort's natural trajectory was one of subsequent improvement. Therefore, the current population is likely to have included a sizeable proportion of patients who had myocardial stunning which improved after prompt revascularization and comprehensive medical therapy. In this context, empagliflozin could not modify progressive adverse left ventricular remodelling.

In addition to a low LVEF, other population characteristics suggested a cohort with a potential risk of progressive adverse remodelling. Why, therefore, did we not see evidence of deterioration in left ventricular volumes and function? The first reason is that the majority of our cohort underwent prompt reperfusion therapy which limits infarct size and reduces the risk of progressive adverse remodelling. Although the mean infarct size was substantial at baseline (36% of myocardial mass), we observed a 10% absolute reduction in infarct size and a 9 g/m^2^ reduction in LVMI over follow‐up in the overall trial cohort. These data suggest that there was a substantial degree of myocardial oedema at baseline that may have contributed to a high prevalence of transient myocardial stunning. As noted above, the natural evolution of ventricular volumes and function in these patients is one of improvement and the majority of this happens within the first 14 days.^30^ Whether we would have seen a different pattern of remodelling changes (and potentially treatment effect) had we recruited patients with evidence of persistent systolic dysfunction at a later time following acute MI is unknown. The second reason potentially contributing to the lack of progressive remodelling is the high number of patients who received pharmacological agents known to prevent or attenuate this process. At randomization, almost all patients were on an ACE inhibitor or ARB and over 80% were prescribed a beta‐blocker. The general pattern of improvement in left ventricular volumes and function seen in the current study is broadly similar to that in the echocardiography substudy of the PARADISE‐MI (Prospective ARNI Versus ACE Inhibitor Trial to Determine Superiority in Reducing Heart Failure Events After Myocardial Infarction) where the mean change in LVESVI, LVEDVI and LVEF in the ramipril group was −2.6 ml/m^2^, +2.9 ml/m^2^ and +6.6%, respectively.^18^ Taken together, the data from these two trials suggest that in the modern reperfusion era with the high use of neurohormonal antagonists, the majority of patients do not experience progressive adverse remodelling in the months following acute MI.

The neutral findings of this trial should be seen in the context of previous trials that have examined the effect of SGLT2 inhibitors in patients following MI. The EMMY (EMpagliflozin in patients with acute MYocardial infarction) trial randomized 476 patients to empagliflozin or placebo within 72 h of PCI for an acute MI with creatine kinase >800 IU/L or a troponin >10 times higher than the upper local laboratory limit of normal.^31^ There was no eligibility requirement for either elevated left ventricular volumes or low LVEF. In EMMY, empagliflozin reduced the primary endpoint of NT‐proBNP, increased LVEF measured using echocardiography by 1.5%, and reduced both LVESVI and LVEDVI by 3.2 ml/m^2^ and 3.9 ml/m^2^, respectively. Several features point to the EMMY cohort being at a lower risk of progressive adverse remodelling than that recruited in EMPRESS‐MI; in EMMY the ventricular volumes were almost half of those in EMPRESS‐MI, LVEF was substantially higher (49% vs. 36% in the placebo group) and baseline troponin T and NT‐proBNP were lower than in EMPRESS‐MI. Indeed, in the recent EMPACT‐MI (Study to Evaluate the Effect of Empagliflozin on Hospitalization for Heart Failure and Mortality in Patients with Acute Myocardial Infarction) trial, the rate of heart failure hospitalization in patients with an LVEF >45% was around a third of that of those with LVEF <35%.^32^ Despite this, the pattern of remodelling was divergent between EMMY and EMPRESS‐MI; in EMMY there was evidence of progressive adverse remodelling with an increase in LVESVI and LVEDVI in the placebo group by 2.3 ml/m^2^ and 7.1 ml/m^2^, respectively. Why there is such an apparent discordance between the trajectory of ventricular remodelling in the two trials (and PARADISE‐MI) is unclear.^18^, ^31^ The background use of evidence‐based pharmacologic therapy was similar and other characteristics known to be associated with the risk of progressive adverse remodelling and risk of incident heart failure such as age, sex and a history of diabetes were similar between EMMY and EMPRESS‐MI.^33^, ^34^ One potential explanation is that there was a substantial difference between the trials in the time from MI to the assessment of LVEF (potentially resulting in less reversible myocardial stunning in EMMY), however this was not recorded in EMMY. Given the presence of a significant treatment effect of empagliflozin on attenuating progressive remodelling in EMMY, and the absence of such an effect in EMPRESS‐MI, then it is possible that empagliflozin has favourable effects on left ventricular structure and function when added to standard of care in patients in whom the natural history is one of progressive adverse remodelling. However, that clinical course and treatment effect was not observed in EMPRESS‐MI.

In EMPACT‐MI, empagliflozin reduced the number of first and total heart failure hospitalizations as well as adverse events of heart failure following an acute MI.^23^, ^24^ The EMPACT‐MI population (*n* = 6522) was very similar to the population enrolled in the current trial (LVEF <45% and/or congestion enrolled within 14 days of acute MI).^23^ Our expectation was that the reduction in incident heart failure in EMPACT‐MI was primarily due to empagliflozin reducing left ventricular volumes and preventing progressive adverse remodelling. The follow‐up in EMPRESS‐MI (24 weeks) was substantially shorter than that in EMPACT‐MI (17.9 months), therefore we cannot discount that longer‐term treatment with an SGLT2 inhibitor may have a favourable remodelling effect. However, the lack of a signal of a beneficial remodelling effect of empagliflozin in the current trial suggests that alternative non‐remodelling mechanisms of action may underlie the benefits seen in EMPACT‐MI which were apparent early during treatment. Numerous alternative mechanisms of the cardiorenal benefits of SGLT2 inhibition have been proposed.^35^ Indeed in the present study, we observed changes in biomarkers (uric acid, haematocrit, and body weight) that are consistent with the known effects of empagliflozin.^36^, ^37^ Further research is therefore required to understand what drives the residual risk of the development of heart failure in high‐risk patients following acute MI and to identify novel treatment targets (e.g. pro‐inflammatory pathways) that are independent of the process of ventricular remodelling.^38^ In DAPA‐MI (Dapagliflozin in Myocardial Infarction without Diabetes or Heart Failure), dapagliflozin improved cardiometabolic outcomes using a win ratio analysis method, but not the composite of cardiovascular death or heart failure hospitalization. The DAPA‐MI population was much lower risk than EMPRESS‐MI or EMPACT‐MI. Patients with any impairment in left ventricular systolic function (regional or global) were eligible (LVEF <50% in 73% of the study population) and type 2 diabetes mellitus was an exclusion. Our remodelling data are therefore less applicable to DAPA‐MI‐type patients.^25^

Although we did not observe progressive adverse remodelling overall in EMPRESS‐MI, a small proportion of patients did have an increase in ventricular volumes over follow‐up. This therefore raises the challenge of how to best identify these patients who are at the highest risk of the development of heart failure and who may stand to benefit the most from additional targeted anti‐remodelling therapy. The majority of ongoing trials are using the traditional enrichment criteria of an anterior MI and low LVEF.^38^ The data from EMPRESS‐MI suggests that these criteria alone are unlikely to identify patients at high risk of progressive adverse remodelling in the context of contemporary pharmacological and reperfusion therapy. Patients with a reduction in LVEF and an infarct characterized by microvascular obstruction and intramyocardial haemorrhage are at the highest risk of adverse outcomes and adverse remodelling compared to patients with a low LVEF without these infarct features.^39^, ^40^ Indeed in the present trial, the mean change in LVESVI and LVEDVI over follow‐up in patients with microvascular obstruction and intramyocardial haemorrhage (present in approximately half of the cohort) was −1 ml/m^2^ and +9.1 ml/m^2^, respectively, compared to −18 ml/m^2^ and −11 ml/m^2^ in those without microvascular obstruction. It is perhaps, therefore, time to move on from an era of relying on infarct location and ventricular function and volumes to identify high‐risk patients to one focused on detailed infarct characterization in addition to a reduction in LVEF to identify patients who are at the highest risk of adverse outcome following acute MI. There are challenges to this approach, such as the current requirement for cardiovascular MRI for tissue characterization, however, efforts should be made to identify potential alternative strategies to detect intramyocardial haemorrhage such as novel circulating biomarkers and echocardiography‐based tissue characterization.^41^, ^42^

### Limitations

Several limitations must be highlighted. Patients only received treatment for 24 weeks, and a remodelling effect may be seen to continue over a longer period.^43^ The SD of the change in LVESVI was higher than we had initially powered for. However given that there was almost no between‐group difference, a larger sample size would have been unlikely to show any clinically meaningful change. The majority of patients recruited presented with a STEMI, therefore, the applicability of the results of the EMPRESS‐MI trial to patients with NSTEMI is limited.

## Conclusion

In patients with left ventricular systolic dysfunction after an acute MI treated with contemporary reperfusion and medical therapy, the addition of empagliflozin to standard care did not have any effect on improving left ventricular volumes or function compared with placebo. We observed that the majority of patients did not display features of progressive adverse remodelling over 24 weeks.

## Supporting information


**Appendix S1.** Supporting Information.
