# Supplementary material for: Empagliflozin to prevent worsening of left ventricular volumes and systolic function after myocardial infarction (EMPRESS‐MI)
Source: Eur J Heart Fail. 2024 Dec 15;27(3):566–76. doi: 10.1002/ejhf.3560 (PMC11955320; doi:10.1002/ejhf.3560)
Supplement: Supplementary file 1 — Appendix S1. Supporting Information. [file EJHF-27-566-s001.docx]

Empagliflozin to prevent worsening of left ventricular volumes and systolic function after myocardial infarction (EMPRESS-MI)

**Supplemental Material**

# Supplemental Methods

## Trial Management Committees, Groups and Individuals

Trial Steering Committee (TSC): The TSC provided overall supervision of the trial and ensured that it was conducted in accordance with the principles of Good Clinical Practice (GCP) and the relevant regulations. The TSC agreed any substantial protocol amendments and provided advice to the investigators on all aspects of the trial.

TSC members: Professor Roy S. Gardner (TSC Chairperson), Professor Colin Berry, Dr Jaclyn Carberry, Mrs Liz Coyle, Dr Kieran F. Docherty, Dr Rachel Myles, Mrs Joanne O’Donnell, Dr Rajan K Patel, Dr Maureen Travers.

Independent data monitoring committee (IDMC): The IDMC responsibilities were to protect the safety of patients recruited to the trial, advise the TSC and co-sponsors if it was safe and appropriate to continue with the study, examine information provided by the Glasgow Clinical Trials Unit on study recruitment, adverse events and outcomes and provide recommendations for the Project Office to forward to the TSC, ethics committees, regulatory bodies, study Co-Sponsors, and the Funder.

IDMC members: Professor Jesse Dawson (IDMC Chairperson), Dr Caroline Coats, Professor Paul Kalra, Professor Jim Lewsey.

Trial Management Group (TMG): The trial was coordinated from National Health Service Greater Glasgow & Clyde (NHS GG&C) by the EMPRESS-MI TMG. The TMG consisted of the Principal Investigator, Chief Investigator and Lead Co-Investigator, trial manager and representatives from the Glasgow CTU, NHS GG&C and The University of Glasgow. The group monitored all aspects of the conduct and progress of the trial and ensured that the protocol was adhered to.

TMG members: Professor Colin Berry (Chief Investigator), Dr Kieran F. Docherty (Lead-Investigator), Professor Mark C. Petrie (Co-Investigator), Dr Katriona Brooksbank (Project Manager).

Robertson Centre for Biostatistics staff: Professor Alex McConnachie (Trial Statistician), Ms Bethany Stanley, Mairi Warren, John McHugh, Robbie Wilson.

Study Co-Sponsor staff: Dr Marc Jones (Pharmacovigilance), Dr Samantha Carmichael and Dr Elizabeth Douglas (Investigational Medicinal Product Management), Dr Maureen Travers and Dr Alison Hamilton (Co-Sponsor contacts for NHS GG&C), Dr Debra Stuart (Co-Sponsor contact for the University of Glasgow), Amanda Lynch (Monitor), Emma Moody (Monitor), Sheila McGowan (Monitor), Margaret Fegan (Monitor).

## Cardiovascular magnetic resonance imaging analysis

Scans were pseudo-anonymised and analyzed by a single operator (M.M.Y.L.) for the purposes of primary and secondary outcomes. The baseline and 24-week scans were analyzed in pairs to reduce intra-observer variability, and the operator was blinded to treatment allocation. Ventricular volumes were measured using the commercially available software package (Circle CVI42 Version 5.14.2, Circle Cardiovascular Imaging, Canada) using standard techniques according to the Society for Cardiovascular Magnetic Resonance.^43,44^ Steady state-free precession short-axis cine images from the mitral valve plane through to the apex were used to calculate ventricular volumes. Specifically, ventricular volumes were calculated by tracing the endocardial border (excluding papillary muscle and trabeculations) in end-systole and end-diastole. Basal left ventricular slice was defined as the most basal slice with >50% myocardium present. Left ventricular outflow tract volume was included in volumetric analysis. End-diastole was defined as the frame in which the blood pool of the mid-ventricular slice was at its largest, and end-systole was defined as the frame in which the blood pool of the mid-ventricular slice was at its smallest. Values for both volumes were indexed by body surface area (BSA), measured at the time of the scan and calculated using the Mosteller formula. Left ventricular ejection fraction (%) was then calculated within the software package (left ventricular ejection fraction = (left ventricular end-diastolic volume – left ventricular end-systolic volume)/ left ventricular end-diastolic volume * 100). Left ventricular mass was calculated as the total difference between the inner and outer circumferences of the left ventricular myocardium in end-diastole, multiplied by the myocardial density (1.05 g/cm^3^), indexed to BSA. Left atrial volume was calculated using the biplane area-length method by manually tracing the left atrium endocardial volume in end-systole. Infarct size, measured in mass and as a percentage of myocardium, was calculated by manually drawing around the epicardial and endocardial border of the late enhancement short axis images, then drawing an area of interest in normal myocardium (180 degrees from area of infarction). An auto-threshold of 5 standard deviations from this normal myocardium was used to identify areas of late enhancement. Microvascular obstruction was defined as a hypointense core within the hyperintense infarct core on late gadolinium enhancement imaging, and was included in the measurement of the infarct size.^45^ Intramyocardial haemorrhage was defined as a region of reduced T2* signal intensity <20 ms within the infarcted area.^45^

**Table S1: Inclusion and exclusion criteria for the EMPRESS-MI trial**

| Inclusion criteria |
| --- |
| Male or female ≥18 years of age |
| Informed consent |
| Diagnosis of a type 1 acute myocardial infarction meeting the Fourth Universal Definition of Myocardial Infarction (ST-elevation myocardial infarction or non-ST-elevation myocardial infarction) |
| Left ventricular ejection fraction <45% as measured by MRI performed ≥12 hours and ≤14 days following hospital admission with an acute type 1 myocardial infarction. For patients with an in-hospital myocardial infarction as qualifying event, randomization must still occur within 14 days of hospital admission |
| eGFR ≥30 mL/min/1.73m^2^ at the time of randomization (calculated using the MDRD formula) |
| Exclusion criteria |
| Inability to give informed consent e.g., due to significant cognitive impairment |
| Diagnosis of chronic heart failure with reduced ejection fraction prior to admission with acute myocardial infarction |
| Systolic blood pressure <90 mmHg at randomization measured after 5 min in a supine or sitting position |
| Cardiogenic shock or use of intravenous inotropes in last 24 hours before randomization |
| Coronary artery bypass graft planned at time of randomization |
| Type 2 acute myocardial infarction |
| Any current severe (stenotic) valvular heart disease |
| Diagnosis of Takotsubo cardiomyopathy |
| Type 1 diabetes mellitus |
| History of ketoacidosis |
| Pacemaker, implantable cardioverter defibrillator or cardiac resynchronization therapy device |
| Permanent or persistent atrial fibrillation |
| Enrolment in another randomized clinical trial involving medical or device-based interventions (co-enrolment in observational studies is permitted) |
| Currently pregnant, planning pregnancy, or currently breastfeeding |
| History of allergy to SGLT2 inhibitor |
| Current or planned use of an SGLT2 inhibitor at time of randomization |
| Active genital tract infections |
| Anyone who, in the investigators’ opinion, is not suitable to participate in the trial for other reason |
| Contra-indication to contrast-enhanced MRI i.e., claustrophobia, metallic foreign object unsuitable for MRI |

Abbreviations: MRI, magnetic resonance imaging; eGFR, estimated glomerular filtration rate; MDRD, modification of diet in renal disease; SGLT2, sodium-glucose cotransporter 2.

**Table S2: Schedule of assessments**

| **Study Procedure** | **Screening**  **(≥12 hours and ≤14 days after acute myocardial infarction)** | **Visit 1 -**  **Randomization**  **(Day 0 - ≥12 hours and ≤14 days after acute myocardial infarction)** | **Visit 2**  **(Week 2±4 days)** | **Visit 3**  **(Week 12±7 days)** | **Visit 4**  **(Week 18±7 days)** | **Visit 5**  **(Week 24±4 weeks)** |
| --- | --- | --- | --- | --- | --- | --- |
| Face-to-face visit | X | X |  | X |  | X |
| Telephone visit |  |  | X |  | X |  |
| Review inclusion/exclusion criteria | X | X |  |  |  |  |
| Echocardiogram | X* |  |  |  |  |  |
| Obtain informed consent | X |  |  |  |  |  |
| Cardiovascular and renal MRI | X† |  |  |  |  | X |
| Physical examination |  | X |  |  |  | X |
| Medical history |  | X |  |  |  |  |
| Medication history |  | X | X | X | X | X |
| Vital signs (Blood pressure/Heart rate) | X | X |  | X |  | X |
| 12 Lead ECG |  | X |  |  |  |  |
| Urine sample |  | X |  | X |  | X |
| Venepuncture (FBC/urea and Electrolytes/liver function tests/HbA1c) | X‡ | X |  | X |  | X |
| Venepuncture (Biomarker analysis) |  | X |  | X |  | X |
| EQ-5D-5L questionnaire |  | X |  |  |  | X |
| Pregnancy testing in WOCBP | X |  |  | X |  | X |
| IMP dispensing |  | X |  | X |  |  |
| Adverse event reporting |  |  | X | X | X | X |
| Study completion |  |  |  |  |  | X |

*Echocardiography performed by the clinical care team was reviewed for purposes of screening.

†In eligible patients

‡Results from the most recent local laboratory test within the preceding 24 hours were used. If not available then performed by investigator who is part of the clinical care team.

Abbreviations: MRI, magnetic resonance imaging; ECG, electrocardiogram; EQ-5D-5L, EuroQol 5-Dimension 5-Level; FBC, full blood count; HbA1c, glycated haemoglobin, WOCBP, women of childbearing potential; IMP, investigational medicinal product.

**Table S3: Medication used during trial follow-up**

|  | **Randomization** | | **Visit 2 (Week 2±4 days)** | | **Visit 3 (Week 12±7 days)** | | **Visit 4 (Week 18±7 days)** | | **Visit 5 (Week 24±4 weeks)** | |
| --- | --- | --- | --- | --- | --- | --- | --- | --- | --- | --- |
|  | **Empagliflozin**  **n=51** | **Placebo**  **n=53** | **Empagliflozin**  **n=50** | **Placebo**  **n=53** | **Empagliflozin**  **n=50** | **Placebo**  **n=53** | **Empagliflozin**  **n=50** | **Placebo**  **n=53** | **Empagliflozin**  **n=50** | **Placebo**  **n=53** |
| Aspirin, n (%) | 51 (100) | 53 (100) | 50 (100) | 53 (100) | 47 (94.0) | 47 (88.7) | 46 (92.0) | 46 (86.8) | 44 (88.0) | 45 (84.9) |
| P2Y12 inhibitor, n (%) | 51 (100) | 53 (100) | 50 (100) | 53 (100) | 50 (100) | 52 (98.1) | 49 (98.0) | 51 (96.2) | 46 (92.0) | 47 (88.7) |
| Anticoagulation, n (%) | 3 (5.9) | 6 (11.3) | 7 (14.0) | 8 (15.1) | 6 (12.0) | 8 (15.1) | 6 (12.0) | 8 (15.1) | 6 (12.0) | 8 (15.1) |
| Statin, n (%) | 50 (98.0) | 53 (100) | 49 (98.0) | 53 (100) | 49 (98.0) | 53 (100) | 49 (98.0) | 53 (100) | 49 (98.0) | 53 (100) |
| ACE inhibitor, n (%) | 46 (90.2) | 41 (77.4) | 44 (88.0) | 41 (77.4) | 41 (82.0) | 41 (77.4) | 40 (80.0) | 38 (71.1) | 39 (78.0) | 36 (67.9) |
| Angiotensin receptor blocker, n (%) | 4 (7.8) | 6 (11.3) | 5 (10.0) | 6 (11.3) | 7 (14.0) | 8 (15.1) | 8 (16.0) | 8 (15.1) | 9 (18.0) | 8 (15.1) |
| Angiotensin receptor-neprilysin inhibitor, n (%) | 0 (0) | 0 (0) | 0 (0) | 2 (3.8) | 2 (4.0) | 3 (5.7) | 2 (4.0) | 6 (11.3) | 2 (4.0) | 7 (13.2) |
| Beta-blocker, n (%) | 42 (82.4) | 47 (88.7) | 44 (88.0) | 47 (88.7) | 46 (92.0) | 49 (92.5) | 48 (96.0) | 49 (92.5) | 49 (98.0) | 48 (90.6) |
| Mineralocorticoid receptor antagonist, n (%) | 33 (64.7) | 33 (62.3) | 33 (66.0) | 36 (67.9) | 41 (82.0) | 42 (79.2) | 43 (86.0) | 42 (79.2) | 45 (90.0) | 41 (77.4) |
| Diuretic, n (%) | 15 (29.4) | 15 (28.3) | 15 (30.0) | 19 (35.8) | 13 (26.0) | 18 (34.0) | 13 (26.0) | 17 (32.1) | 13 (26.0) | 14 (26.4) |
| Loop, n (%) | 15 (29.4) | 15 (28.3) | 15 (30.0) | 19 (35.8) | 13 (26.0) | 18 (34.0) | 13 (26.0) | 17 (32.1) | 13 (26.0) | 13 (24.5) |
| Thiazide, n (%) | 0 (0) | 0 (0) | 0 (0) | 0 (0) | 0 (0) | 0 (0) | 0 (0) | 0 (0) | 0 (0) | 1 (1.9) |

Abbreviations: ACE, angiotensin-converting enzyme.

**Table S4: Signs and symptoms of heart failure at trial follow-up**

|  | **Empagliflozin**  **n=50** | **Placebo**  **n=53** |
| --- | --- | --- |
| NYHA class I, n (%) | 42 (84.0) | 39 (73.6) |
| NYHA class II, n (%) | 6 (12.0) | 11 (20.8) |
| NYHA class III, n (%) | 1 (2.0) | 3 (5.7) |
| NYHA class VI, n (%) | 1 (2.0) | 0 (0) |
| Third heart sound, n (%) | 0 (0) | 0 (0) |
| Pulmonary crepitations, n (%) | 0 (0) | 0 (0) |
| Pleural effusion, n (%) | 0 (0) | 0 (0) |
| Peripheral edema, n (%) | 2 (4.0) | 3 (5.7) |

Abbreviations: NYHA, New York Heart Association.**Table S5: Categories of left ventricular ejection fraction at trial follow-up**

|  | **Empagliflozin**  **n=48** | **Placebo**  **n=52** |
| --- | --- | --- |
| LVEF <45%, n (%) | 32 (66.7) | 30 (57.7) |
| LVEF ≥45%-<50%, n (%) | 5 (10.4) | 13 (25.0) |
| LVEF ≥50%, n (%) | 11 (22.9) | 9 (17.3) |

Abbreviations: LVEF, left ventricular ejection fraction.

**Table S6: Change in non-indexed cardiovascular MRI values with empagliflozin or placebo from baseline at 24 weeks**

|  | **Empagliflozin** | | | | **Placebo** | | | | **Between-group difference (95% CI) *** | **P Value** |
| --- | --- | --- | --- | --- | --- | --- | --- | --- | --- | --- |
|  | **n** | **Baseline** | **24 weeks** | **Change** | **n** | **Baseline** | **24 weeks** | **Change** |  |  |
| LVESV, mL | 48 | 131.8 (45.4) | 113.8 (40.9) | -18.0 (28.1) | 52 | 123.3 (30.4) | 109.1 (40.0) | -14.2 (31.6) | -2.1 (-13.3, 9.2) | 0.71 |
| LVEDV, mL | 48 | 196.1 (56.0) | 194.4 (49.3) | -1.8 (34.5) | 52 | 191.4 (36.4) | 192.7 (50.8) | 1.3 (37.3) | -2.5 (-15.9, 11.0) | 0.72 |
| LAV, mL | 48 | 69.1 (29.7) | 73.8 (30.3) | 4.7 (28.4) | 52 | 70.8 (21.7) | 77.2 (26.7) | 6.4 (27.4) | -2.6 (-12.6, 7.4) | 0.61 |
| LVM, g | 48 | 125.2 (37.7) | 103.4 (27.3) | -21.8 (17.0) | 52 | 116.9 (27.6) | 99.8 (22.0) | -17.1 (14.1) | -2.3 (-6.8, 2.1) | 0.30 |

Data presented as mean (SD) unless otherwise stated. Results reported for those with data available at baseline and 24 weeks. *Calculated using a linear model adjusted for randomized treatment, baseline value of the outcome, use of diuretics at baseline and diabetes status. Between-group differences are reported as adjusted mean differences (95% CI). Abbreviations: CI, confidence interval; MRI, magnetic resonance imaging; LAV, left atrial volume; LVEDV, left ventricular end-diastolic volume; LVESV, left ventricular end-systolic volume; LVM, left ventricular mass.

**Table S7: Adverse events of special interest**

| **Safety outcome, n (%)** | **Empagliflozin**  **(n=51)** | **Placebo**  **(n=54)** |
| --- | --- | --- |
| Creatinine ≥ 2-fold increase from baseline and above the upper limit of normal | 0 (0) | 1 (1.9) |
| Hepatic injury* | 2 (3.9) | 0 (0) |
| Diabetic ketoacidosis | 0 (0) | 0 (0) |
| Major hypoglycemic event | 0 (0) | 0 (0) |
| Lower limb amputation | 0 (0) | 0 (0) |

n = number of patients with event (%). *Hepatic injury defined by the following alterations of liver parameters after randomization at Visit 1: Elevation of aspartate aminotransferase (AST) and/or alanine transaminase (ALT) ≥ 3 fold upper limit of normal (ULN) combined with an elevation of total bilirubin ≥2 fold ULN measured in the same blood draw sample, isolated elevation of AST and/or ALT ≥ 5 fold ULN irrespective of any bilirubin elevation.

**Table S8: Change in systolic blood pressure and heart rate with empagliflozin or placebo from baseline at 24 weeks**

|  | **Empagliflozin** | | | | **Placebo** | | | | **Between-group difference (95% CI) *** | **P Value** |
| --- | --- | --- | --- | --- | --- | --- | --- | --- | --- | --- |
|  | **n** | **Baseline** | **24 weeks** | **Change** | **n** | **Baseline** | **24 weeks** | **Change** |  |  |
| Systolic blood pressure, mmHg | 49 | 113.9 (14.9) | 129.7 (18.4) | 15.8 (20.6) | 53 | 113.8 (16.1) | 131.0 (22.3) | 17.3 (20.3) | -1.0 (-8.2, 6.2) | 0.78 |
| Diastolic blood pressure, mmHg | 49 | 68.4 (10.4) | 70.5 (7.9) | 2.1 (9.7) | 53 | 66.7 (8.1) | 69.5 (9.9) | 2.8 (10.3) | 0.5 (-2.8, 3.7) | 0.77 |
| Heart rate, bpm | 49 | 79.7 (13.2) | 66.6 (9.2) | -13.1 (13.2) | 53 | 74.8 (12.6) | 64.8 (10.3) | -10.0 (14.5) | 1.0 (-2.7, 4.8) | 0.58 |

Data presented as mean (SD). Results reported for those patients with data available at baseline and 24 weeks. *Calculated using a linear regression model adjusted for randomized treatment, baseline value of the outcome, use of diuretics at baseline and diabetes status.

Abbreviations: bpm, beats per minute; CI, confidence interval; mmHg, millimeters of mercury.

**Figure S1: CONSORT diagram**

**
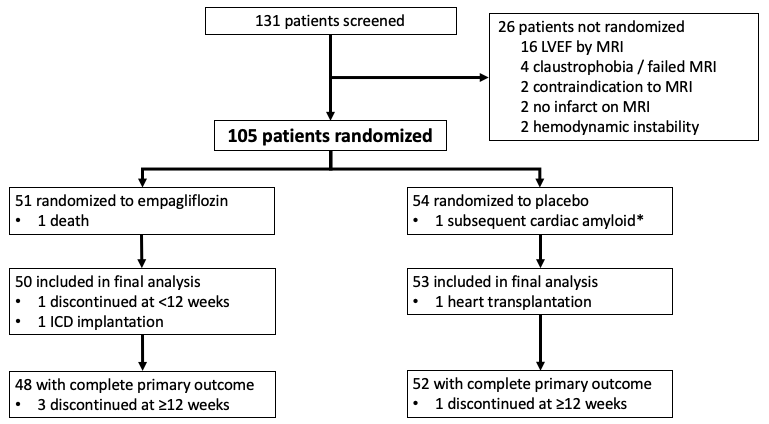
**

*This participant had a subsequent diagnosis of cardiac amyloid following randomization. After discussion with the Trial Steering Committee and Trial Management Group, and prior to unblinding, this randomization was classified as a major protocol deviation, as the presence of cardiac amyloid would have met the exclusion criterion “Anyone who, in the investigators’ opinion, is not suitable to participate in the trial for other reason”.

Of the 16 patients who did not meet inclusion criteria by MRI LVEF, 10 had an LVEF ≥45%.

6 patients discontinued the study drug early; 4 in the empagliflozin group and 2 in the placebo group.

Within the empagliflozin group, 1 stopped with <12 weeks of drug treatment, due to recurrent genital infections, and therefore did not attend for follow-up MRI as per study protocol. This patient attended for all other outcome analysis. 3 discontinued therapy with at least 12 weeks of drug exposure and attended for follow-up MRI. The reasons for discontinuation were: 1 patient concern about side effects, 1 recurrent genital tract infection, 1 symptomatic heart failure development. 1 patient in the empagliflozin group received an ICD after randomization and therefore did not attend for follow-up MRI, but remained on therapy and attended for all other outcome analysis.

Within the placebo group, 1 stopped with <12 weeks of drug treatment, due to receiving a heart transplant, and therefore did not attend for follow-up MRI as per study protocol. This patient attended for all other outcome analysis. 1 discontinued therapy with at least 12 weeks of drug exposure, due to symptomatic heart failure development, and attended for follow-up MRI.

Abbreviations: MRI, magnetic resonance imaging; ICD, implantable cardioverter defibrillator; LVEF, left ventricular ejection fraction.
